# Microfluidics‐based 3D cell culture models: Utility in novel drug discovery and delivery research

**DOI:** 10.1002/btm2.10013

**Published:** 2016-07-05

**Authors:** Nilesh Gupta, Jeffrey R. Liu, Brijeshkumar Patel, Deepak E. Solomon, Bhuvaneshwar Vaidya, Vivek Gupta

**Affiliations:** ^1^ Neofluidics LLC, Research and Development Wing San Diego CA 92121; ^2^ DS Laboratories, Inc., Research and Development Pompano Beach FL 33064; ^3^ School of Pharmacy Keck Graduate Institute Claremont CA 91711

**Keywords:** chip, matrix, microfluidics, nanoparticles, spheroid, three‐dimensional culture

## Abstract

The implementation of microfluidic devices within life sciences has furthered the possibilities of both academic and industrial applications such as rapid genome sequencing, predictive drug studies, and single cell manipulation. In contrast to the preferred two‐dimensional cell‐based screening, three‐dimensional (3D) systems have more in vivo relevance as well as ability to perform as a predictive tool for the success or failure of a drug screening campaign. 3D cell culture has shown an adaptive response to the recent advancements in microfluidic technologies which has allowed better control over spheroid sizes and subsequent drug screening studies. In this review, we highlight the most significant developments in the field of microfluidic 3D culture over the past half‐decade with a special focus on their benefits and challenges down the lane. With the newer technologies emerging, implementation of microfluidic 3D culture systems into the drug discovery pipeline is right around the bend.

## 1 Introduction

Cell culture, an alternative to organ culture and in vivo animal models, is an integral part of several ongoing studies pertaining to biomedical research including biochemistry, biology, pharmacokinetics, and pharmacodynamic discovery and development of therapeutic drugs, as well as tissue engineering.^1^ Cell culture models offer an easily accessible, highly reproducible, and reliable mode of investigation with capability of high throughput screening (HTS). Cell culture studies are essential to make a “go/no‐go” decision before proceeding toward further preclinical and human studies.^2^ Human body is a very complex system with multitude of cell types interacting with each other for sharing and propagation of crucial information. The physiological cellular network resembles an electronic circuit of a supercomputer which needs integration and coordination of hundreds and thousands of microcomponents (chips) for calculation and analysis of data. Similarly, a cell, basic unit of tissue, works in a coordinated fashion with other cells to carry out its essential functioning. In diseased conditions, cells start behaving in an abnormal fashion which can be characterized by growth, differentiation, secretion of markers, invasion, migration, or premature death. These abovementioned properties of cells are harnessed to study the effect of drugs, both small and large molecules, and are utilized effectively for discovery and development of new therapeutic molecules. Also, these attributes provide information regarding differences between normal and diseased tissues.

While countless studies delineate the mechanisms of cellular growth and pathogenesis, the actual environment inside the tissues still remains more of an enigma. Broadly speaking, cells grow and arrange themselves in a three‐dimensional (3D) format and are elliptical with 100% of their surface area exposed to other cells for vital processes such as cell‐to‐cell signaling, gene/protein expression, response to external stimuli and growth cycle to name a few.^1^ Cell culture, after its discovery in 1907, has observed and underwent many significant changes which has led to a near‐perfect modeling of human system.^1^ In traditional culture, cells are grown on a flat surface as a monolayer. Culture flasks, wells, and Petri dishes are commonly used to grow them by providing a medium as a source of nutrition at physiological temperature (37 °C). Medium is enriched with serum and glutamine to boost growth and, a cocktail of antibiotics to prevent infections. Depending on the doubling time, cells acquire confluence after a certain period of time and after that they are subcultured to avoid competition among themselves for nutrition. This is done by detaching them from the surface using trypsin and/or ethylenediamine tetraacetic acid (EDTA) and reseeding into new flasks for the further growth of cell line. This protocol is usually termed as “*Two‐dimensional (2D) Culture*.”

**Figure 1:**
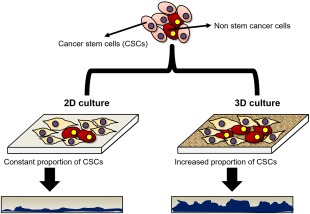
Comparison of 2D and 3D cell culture. Cells grown on conventional 2D surfaces adopt a typical flattened morphology covering mainly *x‐y* plane and have a reduced height in the vertical *z* plane. In comparison, 3D culture allows more cuboidal morphology and 3D structure, particularly in *z* plane (modified from Ref. 4)

As 2D culture does not mimic the inherent physiological conditions, use of 3D culture systems has come to light to bridge the unfilled gaps.^3^ Cells are a product of their 3D complex matrix‐based environment which facilitates cellular communications and secretions. In vivo, each cell is 100% exposed to the neighboring cell which is not present in 2D‐based culture and hence it limits the predictive accuracy during the experimental and clinical studies. Recently, 3D culture has gained widespread attraction because of its several advantages over 2D culture (Figure 1).

## 2 Advantages of 3D cell culture over 2D cell culture

The 2D and 3D cell cultures can be compared on the basis of several features which lead to difference in effects including cellular morphology, phenotype, metabolic activity, and cellular functionality.

### 2.1 Morphology

Cells in 2D culture are typically flat with average thickness of ∼3 µm whereas in 3D culture, cells are ellipsoids having dimensions of ∼10–30 µm. Cells grown as monolayers do not show altered morphology as observed in diseased conditions such as cancer or inflammation. For example, 3D culture shows clear differences in the morphology, alignment, integrity, and polarization of human bladder carcinoma cells as compared to 2D culture of the same cells.^5^ Human retinal cells show more neurite extension in 3D.^6^ Vascular smooth muscle cells show more prominent stress fiber formations and focal adhesions in 3D but not in 2D culture.^7^ With an added dimension, 3D cell culture offers a more applicable morphological understanding of the cellular environment providing a deeper insight into the cellular responses and the associated changes to their structure.

### 2.2 Differentiation

Cellular differentiation is well characterized and evidenced in 3D culture. In contrast, 2D culture is not efficient in predicting the differentiation. As shown by Farrell et al., modulation of osteogenesis of adult rat mesenchymal stem cells could be clearly seen in 3D culture, as marked by expression of collagen type I which was not evident when the cell culture was performed in 2D manner.^8^ Also, markers indicative of differentiation and other parameters such as duration, phenotypic changes, state of nondifferentiation can be easily visualized under microscope in 3D culture.^9^ While 2D methods have been optimized for most conventional studies; this tool falls short when understanding the progression of cellular differentiation. Due to enhanced in vivo relevance that 3D culture offers, this new multifaceted tool allows a more comprehensive study to understand the nascent cellular behavior.

### 2.3 Viability

Cells in 2D culture are less viable and more susceptible to apoptosis than in 3D culture. Cells behave differently in 3D culture because of more prominent cell‐to‐cell interactions.^10^, ^11^ Smooth muscle cells are more viable in 3D systems, even under suboptimal conditions (depletion of nutrients).^10^ Some cartilage cells show differences in growth kinetics when cultured in 3D systems.^12^ Also, cancer cells show more differences related to cell death in response to drugs in 2D/3D systems.^13^ The 3D culture promotes more interactions among cells allowing them to remain healthier in suboptimal conditions.

### 2.4 Response to stimuli

As there are several types of stimuli either triggered by adjacent cells or external factors, cells respond to them in different ways when cultured differently. Lin et al. showed that 3D culture showed no effect on human MCF‐10A cell morphology and sensitivity after radiation exposure, while they were found to be sensitive in 2D culture.^4^ In another study, Merwin et al. outlined that TGF‐β did not exert any antiproliferative effects on human endothelial cells in 3D systems.^14^ Osteoblasts, when cultured in 2D system, showed less proliferation in response to shear stress as compared to 3D culture.^15^ With the addition of 3D cell culture to the life sciences toolbox, it has been easier to differentiate between the normal and stimuli based responses of cells.

### 2.5 Drug metabolism

Cells metabolize drugs and secrete metabolic products in a far distinctive manner when cultured in 3D systems. H358 cells showed variable cytotoxicity in response to drugs such as paclitaxel, doxorubicin, and vinorelbine in 3D culture as compared to 2D system.^16^ Elkayam et al. demonstrated that hepatocytes secrete more urea and albumin and show enhanced resistance in response to drugs in 3D culture. Also, they showed increased CYP p‐450 activities in response to addition of drugs.^17^ Cancer cell lines such as MCF‐7, Lovo, and PC‐3 showed increased/decreased chemosensitivity in 3D culture utilizing the large and porous biodegradable microparticles as matrix which suggest the significant roles of cellular architecture, variation in phenotypes, and extracellular matrix (ECM) barrier to drug transport phenomena.^18^, ^19^

### 2.6 Gene expression and protein synthesis

Neuroblastoma cells, when grown in 3D culture model, show altered differential expression of about 1,766 genes, including those relevant to cytoskeleton, ECM, and neurite outgrowth, as compared to 2D culture, differences are attributed to influence of culture material on the gene expression, cell spreading, and neurite growth.^5^ Vascular smooth muscle cells showed twofold increased expression of 77 genes and reduced expression of 22 genes in 3D systems because of less stress fibers formation and focal adhesions in 3D matrix.^6^ Hybridoma cells showed increased production of monoclonal antibodies suggested by reduced apoptosis and resistance to low‐serum environments in 3D fibrous matrix.^12^ MCF‐7 cell line cells showed increased expression of E‐cadherin, catenin and p27 and synthesis of collagen owing to different state of cell adhesion and expression of intercellular adhesion molecules in 3D environment.^18^

### 2.7 Cell functions

Human HepG2 liver cells showed enhanced performance and functional activity in polystyrene scaffold‐based 3D culture.^20^ In 3D culture of bladder carcinoma cell line, RT112, cells demonstrated well developed cell‐cell contacts, a distinct endoplasmic reticulum and marked Golgi apparatus within multicellular spheroid‐like structure.^4^ Bone marrow stem cells showed enabled calcification and increased alkaline phosphatase activity in 3D network of nanofibers which enabled better attachment, proliferation, and osteogenic differentiation.^21^ HER2 over expressing cells were marked with formation of homodimers as opposed to heterodimers in 3D culture which makes them more activated and a switch in signaling pathways close to in vivo systems.^22^

### 2.8 In vivo relevance

Tumors which are characterized with polarized epithelial structures or spheroids with more cell‐to‐cell contacts are more prominent in 3D culture.^23^ In a study by Merwin et al., human endothelial cells demonstrated more tube‐like structures mimicking angiogenesis due to enhanced tight junctions and abluminal basal lamina deposition in 3D cultures.^14^ In another study, rat hippocampal region was shown to have increased neuron/astrocyte ratio conferred due to stability in 3D‐based cultures.^24^ Cow articular cartilage cells showed similar in vivo histology in 3D culture.^11^ Rat olfactory cells maintained their original spindle‐shape morphology in 3D collagen scaffolds which provide suitable environment to maintain their morphology and functional phenotypes.^25^

### 2.9 Proliferation

Increased growth rate of mesenchymal stem cells, osteosarcoma cells, human umbilical vein endothelial cells (HUVEC) and tumor epithelial cells (TEC) cells, and human glioblastoma cells has been reported in 3D fibrous matrix‐based culture models and where they were more protected from shear stress and had lower apoptosis even under nutrient depletion.^9^, ^26^, ^27^ However, human neuroblastoma, breast cancer, sheep bone marrow, rat interior tibialis muscle, and airway smooth muscle cells showed decreased proliferation due to differences in morphology, lower contractile protein expression and basal proliferation in 3D cultures.^28^, ^29^, ^30^

As discussed above, there has been an argument about the correlation of results obtained from 2D cultures and their relevance to in vivo scenarios, which stems from differential behavior of cells in vitro and in vivo. Naturally in human body, the cells grow in a 3D pattern. In addition to interacting with ECM, the cells interact with other cell types as well, which affects a broad range of cellular functions.^31^ Capability to grow cells in a 3D format bridges the gap between in vitro and in vivo conditions, and hence is the most appropriate form of representation of real‐life in vivo scenarios. The 3D culture models have become increasingly relevant to the biomedical research, and are continuously being recommended as a “*must do*” before moving on to the more advanced studies. In the sections below, we will discuss the most common methods used for 3D cell culture, their importance and relevance to biomedical research, and will also try to elaborate on the need for further refinement of the culture models.

## 3 Conventional methods for 3D cell culture

**Figure 2:**
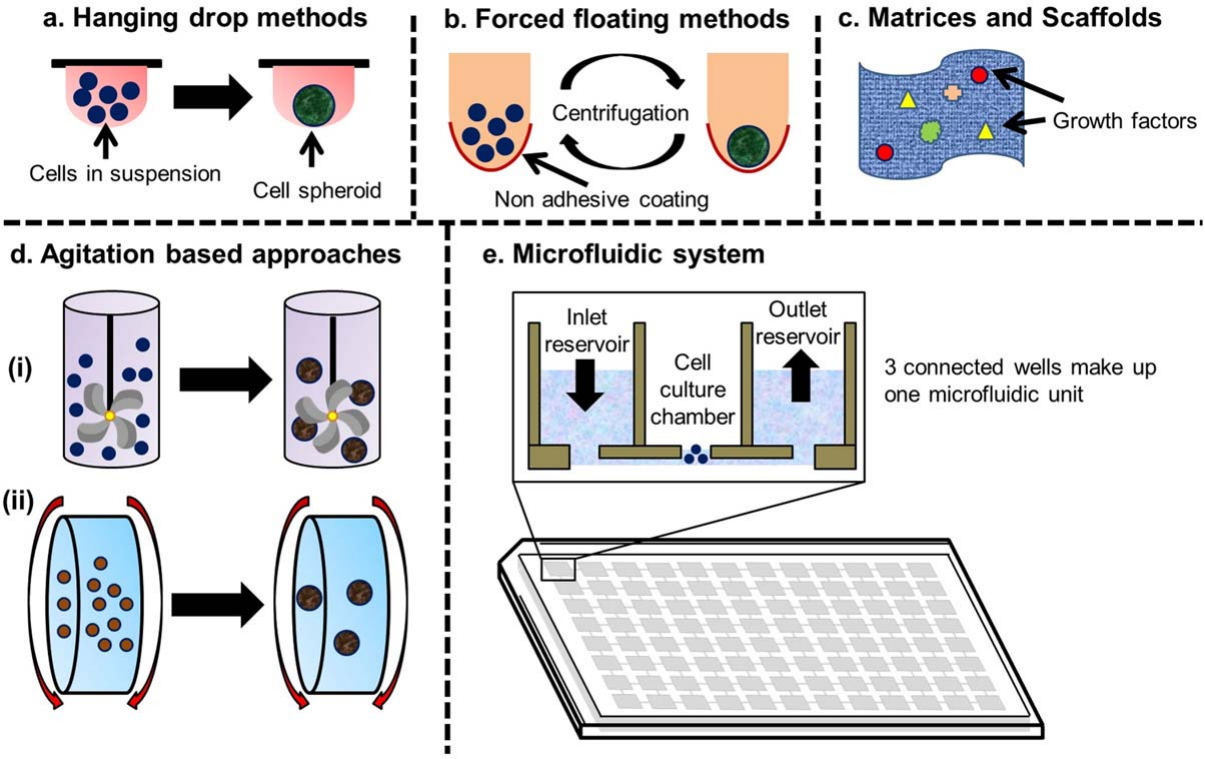
Conventional methods for 3D cell culture. (a) Hanging drop. (b) Forced floating. (c) Matrices and scaffolds. (d) Agitation based approaches, (i) spinner flask and (ii) rotating cell culture bioreactors. (e) Microfluidic systems (modified from Refs. 31, 32, 33, 47, 56)

Several methods have been reported by different researchers to develop 3D cell cultures. These include hanging‐drop method, forced‐floating method, matrices, scaffolds, and agitation based approaches (Figure 2).

### 3.1 Hanging‐drop method

This is a relatively simple and easy to execute method with a reported reproducibility of 100% for producing one 3D spheroid per drop for several cell lines.^32^ As reported by Kelm et al., a small volume (20–50 µl) of single cell suspension at a density of 50–500 cells/well is usually pipetted into wells.^33^ After seeding the cells, the plate/tray is inverted which turns the aliquot into a hanging droplet of cells. The cells concentrate at the tip of the drop and remain in place due to surface tension, and the spheroids are tightly packed and overall homogenous in morphology.^34^ A disadvantage of this method is that larger volume (>50 µl) cannot be used since the surface tension can no longer hold the droplet. Two newer technologies by 3D Biomatrix and InSphero have tried to answer these issues. The 3D Biomatrix demonstrated a 384‐well hanging drop plate which can easily support large‐scale production of spheroids.^35^ InSphero modified the above mentioned plate with “trap” technology which allows easy harvesting of cultured spheroids.^35^

### 3.2 Forced‐floating method

Forced‐float method is a simple yet reproducible method to produce consistent spheroids. Cells grown by this procedure are prevented from attaching to the surface by several modifications, allowing force‐floating and hence promoting cell‐to‐cell interactions.^35^ Ivascu and Kubbies used this method for the rapid production of cancerous and noncancerous spheroids in different types of well plates.^36^ In their method, plates were coated with poly‐hydroxyethylmethacrylate (poly‐HEMA) which prevented adhesion of cells to the surface. Eight types of breast cancer cells were seeded in which some formed tight spheroids while others produced loose aggregates. This problem was solved by adding reconstituted basement membrane to the suspension of cells and, within 24 hr, compact 3D spheroids were formed with enhanced consistency. A 96‐well plate is typically used for this method and sizes of spheroids can be manipulated by simply changing the quantity of seeded cells. Another inexpensive alternative is to use agarose for coating purposes, which also enables long‐term culture (>20 days) of spheroids.^37^ The main concern is time consumed when coating the plates, as the coating polymer needs to be dissolved and autoclaved prior to use.^38^ Few precoated plates such as PrimeSurface, Lipidure, and Sumitomo Bakelite are available in market but it should be noted that these expensive plates increase the overall cost of spheroid production.^39^, ^40^

### 3.3 Matrices and scaffolds

Use of ECM to produce 3D spheroids is a relatively easy method. Sterile ECM is commercially available and can be used to culture the cells; (a) to embed and grow cells within the gel and (b) to grow them on top of the gel.^41^ ECM plays an important role in enabling the cells to perform better communication with other cells and cell‐ECM interaction is vital for proper cellular functions.^42^, ^43^ Various types of ECM are commercially available which helps further in designing the appropriate experiments. BD Biosciences has ECM available as Matrigel which has been extensively used in the production of 3D mammospheres and human hepatocarcinoma cells.^32^, ^44^ Matrigel is composed of tumor‐derived basement membrane proteins such as collagen IV, MMPs, perlecan, entactin, laminin, and growth factors, essential for cell differentiation and propagation of signaling cascade.^43^ Breast tissue is a highly branched yet organized complex structure comprising of epithelial cells.^45^ Culture of MCF‐7 cells in Matrigel showed a stromal structure with better interactions with ECM which helped in enhanced cellular signaling.^32^, ^46^ Some disadvantages associated with ECM‐assisted culture are nonuniformity of spheroids, expensive for large scale productions and batch‐to‐batch variability. These problems were rectified using ECM in an array‐based system which utilizes soft lithography to produce microstructures which acted as wells in/on which cells can be cultured as spheroids.

For scaffold‐based 3D culture, collagen, laminin, alginate, and so forth are used to construct prefabricated scaffolds. These scaffolds consist of a network of fibers through which cells can easily migrate near to other cells and attach.^31^ As the cells divide and grow, they fill the interstitial space between the fibers producing a 3D‐like morphology. Typically, they are known as hydrogels which offer a porous structure which allows prolonged availability of nutrients, drugs, and oxygen necessary for survival along with removal of waste products. This assembly provides appropriate cell culture conditions for better mobility and organization of cells.^31^ Various companies such as GE Healthcare, Solohill, Global Cell Solutions have successfully launched microcarrier beads for 3D culture of cells in bioreactors.^31^ The main disadvantage associated with such technique is the special equipment required for this type of culture.

### 3.4 Agitation‐based approaches

The basic principle of this approach is that a cell suspension is placed into a container while keeping the suspension in motion. Gentle stirring of rotation is used to provide motion to cells. Due to this, cells do not adhere to the walls and form cell‐to‐cell interactions.

1. *Spinner flask bioreactors*: They consist of a container and a stirring element to hold and continuously stir the cell suspension.^47^ Size of the container can be varied and hence spheroids of different sizes can be produced. Medium can be changed periodically to ensure long‐term culture of cells. Motion of culture fluids assists in providing nutrients to cells and subsequent removal of waste products.^46^ Drawbacks associated with spinner systems are altered physiology of cells due to sheer force of stirring bar, requiring a larger amount of culture medium and inconsistency in the sizes of spheroids formed.^48^ These issues can be addressed by first culturing the spheroids in agarose coated wells and transferring to spinner flasks.^49^ Some commercially available spinner assemblies are from companies like Wheaton and Corning.
2. *Rotating cell culture bioreactors*: While the functioning of this system is similar to spinner flasks, the whole container is rotated instead of using a stirrer bar/rod. Initially, when cells are in single cell suspension, the culture chamber is rotated at low speed; however, as the cells begin to form larger aggregates, the speed is increased to maintain the spheroids in suspension. Low sheer force is the main advantage of this system.^50^ While this system is simple, allowing easy handling, and large‐scale and long‐term production of spheroids, there is large variability in the size of the spheroids.^51^ Synthecon provides commercial rotary cell culture systems.
3. *Other bioreactors*: There are few more, although not very popular, bioreactor‐based 3D culture systems such as rotary perfusion and compression bioreactors. Rotary perfusion system allows a continuous feeding of the cell chamber from external media bottle; cells are retained in the chamber by molecular weight cutoff membrane.^52^ Compression bioreactor provides a controllable mechanical and physiological environment for simulating in vivo conditions in vitro, and is generally used in cartilage engineering.^53^

### 3.5 3D bioprinting

Also known as additive manufacturing, 3D printing of biocompatible materials, constituent cells and supporting structures into functional living organs is gaining momentum in the field of drug discovery and research. Although very complex and cumbersome, bioprinted heart, cartilages, bones, skin, and vascular grafts have been employed for transplantation purposes.^54^ Further research is going on to make this process high throughput so as to utilize in the mainstream drug discovery cascade.

## 4 Summary of pitfalls of current 3D culture systems

While 3D cell culture systems offer state‐of‐the‐art technology for enabling drug development and several other applications, there are many unmet needs and gaps which need to be filled to get a universal standardized and validated system.^55^ Applications of 3D culture differ in academia and industries. Academia focusses on biological relevance whereas industries look for more cost‐efficient, automated and easily readout systems. Below are some drawbacks of the currently available 3D culture systems:

1. Existing systems represent static conditions rather than mimicking the biochemically dynamic characteristics of the tissue.
2. There is a risk of transmission of infections or diseases from human‐/animal‐derived materials used to prepare scaffolds.
3. Reproducibility of scaffold‐based culture as there is significant batch‐to‐batch variation.
4. Protocol optimization to isolate proteins from 3D cultured cells is needed.
5. Synthetic scaffolds, PEG‐based, are typically inert in nature and require modifications prior to embedding and cell growth.
6. HTS and processing is difficult as sometimes cells or scaffolds demonstrate autofluorescence.
7. More calibration of scaffold‐based 3D culture systems as there is significant interaction of drugs/molecules with the materials used to prepare scaffolds/matrices.

## 5 Microfabricated methods for 3D culture

Microfluidic technology came into existence in the 1990s and offered a great and more versatile platform for biological applications.^56^ The 3D cell culture techniques, in particular, have been revolutionized by the integration of microfluidic technology. It is also known as Lab‐on‐a‐chip or micro total analysis system and has been used for countless biomedical applications such as in drug discovery and development, toxicity, cell culture, genetic assays, protein studies, intracellular signaling, stem cells, tissue engineering, pathogen detection, to name a few.^57^, ^58^ The microenvironment provided by microfabricated systems are really compatible with those found in vivo. Some significant features which make this technology distinguishable are:

1. Microscale dimensions’ match with the cellular structures and traffic present inside the human body.
2. Chemical gradient can be created to mimic the complex and dynamic 3D network present in real‐life systems.
3. Very cost effective as it utilizes samples in nanoliter volumes and hence reagents are consumed in very less quantities.
4. Substrates used for constructing microfluidic devices (including silicone and biodegradable polymers) are often permeable to oxygen which enables better growth and proliferation of cells in 3D culture.
5. Microfluidics represent a multifaceted technology which can handle several processes at one time such as culture, replenishment of medium, cell detachment, sampling, mixing, capture, and subsequent detection. An ideal 3D culture should promote growth of cells by supplying needed nutrition, moisture, oxygen as well as remove degradation products at the same time. Microfluidic technology offers all these privileges to cell culture in 3D format and has thus revolutionized the field.

**Figure 3:**
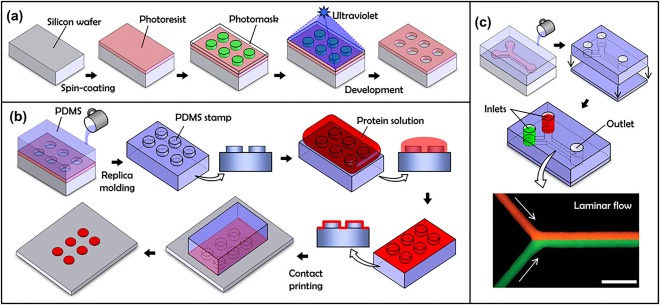
Microfabricated methods to establish 3D culture systems. (a) Photolithography, the core microfabrication technique. (b) Replica molding and microcontact printing. (c) Bonding of microfluidic devices and laminar flow (adapted from Ref. 60)

Different types of microfluidic systems have been used to establish and support 3D culture and have been categorized based on the substrates used to fabricate the microdevices.^59^, ^60^ Fabrication is typically done by photolithography techniques which includes standard Radio Corporation of America (RCA) cleaning, thin film deposition, wet hydrofluoric etching, access hole forming, and chip bonding (Figure 3). Microfabrication is done initially in integrated circuit. Different platforms used for 3D culture in microfabricated devices are discussed below.

### 5.1 Glass‐/silicon‐based systems

Glass‐based systems offer enhanced optical properties which are advantageous in high resolution microscopy. These systems can be used multiple times and in long‐term studies due to capability of glass to provide a well‐defined stable surface with reproducible and reliable electroosmotic flow.^61^ Jang et al. used Tempax glass to prepare a 3D continuous‐perfusion microchip system for culturing osteoblasts. Photolithographic etching method was used to design and synthesize the device, which was further tested for drug screening applications for more than 10 days.^62^ In another study, Lin et al. prepared a similar chip but used indium tin oxide glass which had heating functionality and real time microscopic application.^61^

**Figure 4:**
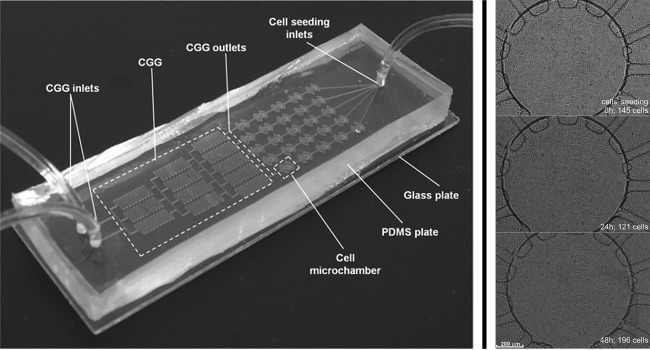
PDMS‐/glass‐based microfluidic system for the culture of A549 cells. This microchip consisted of an integrated concentration gradient generator and was used for cytotoxicity and cell‐splitting experiments (adapted from Ref. 64)

Glass‐based systems have a remarkable property of being impermeable to oxygen, which has been repeatedly utilized to create hypoxic conditions and carrying out cellular studies. Hattersley et al. used this property to culture tissue biopsy samples and measured vascular endothelial growth factor (VEGF) release to compare normal and cancerous tissues.^63^ Recently, polydimethysiloxane (PDMS) have gained popularity and are now the most commonly used form of substrate to prepare microchips. Chudy et al. prepared a hybrid microchip comprising of PDMS and glass with an integrated concentration gradient generator.^64^ This chip was used several times for cytotoxicity and cell splitting experiments (Figure 4).

Silicon‐based systems, on the contrary, are not in wide‐spread use because of their high cost and complicated fabrication procedures. Ling et al. used an SU‐8 mold mask on a silicon wafer for preparing an agarose based system which was used to deliver essential nutrients and oxygen to cells in hydrogels.^65^

### 5.2 Polymer‐based systems

Various polymers such as PDMS, polycarbonate, polystyrene, polymethyl methacrylate (PMMA) have been used as biocompatible substrates for microdevices. Polymer‐based platforms are dominated by PDMS because of its permeability to oxygen and cost effectiveness.^66^, ^67^ Over the past few decades, microfluidic 3D cell culture has adopted several names, depending on the structural differences, such as microwells, microchannels, micropillars, and cell retention chambers.^68^ PDMS has been extensively used to design these devices to facilitate dynamically perfused 3D culture. All of these devices were optimized for the flow of medium and perfusion of oxygen throughout the culture regions which reflected more in vivo like conditions. Typically, there are two ports in the device; (a) one inlet port through which medium is injected to provide the essential growth factors and oxygen; and (b) one outlet port which is used to eject the remaining medium along with metabolic degradation by‐products.^69^ In some advanced devices, medium‐infused channels have been integrated with microwells which enables spatial and temporal investigation of several factors regulating cell differentiation.^70^

Some naturally originated polymers such as agarose, fibrin, and collagens have also been used to create microfabricated cell‐laden devices for 3D culture. Ling et al. cultured multiple cell types including hepatocytes, and AML 12 in an agarose‐based device created by utilizing soft lithography technique.^65^ In this regard, they fabricated 1 cm thick cell‐laden agarose replica molds by cooling a hot solution of agarose to 70 °C. Cell suspension was then mixed and agarose‐cell mixture was poured onto a silicone master for gelation.

### 5.3 Paper‐based 3D culture platforms

**Figure 5:**
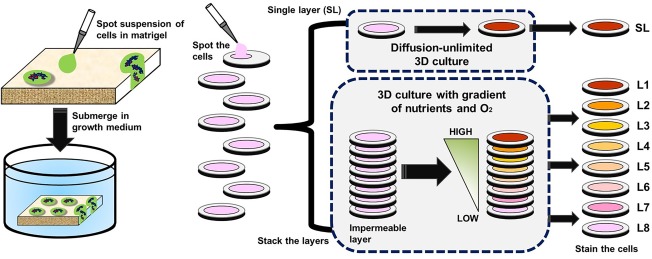
Paper‐based systems for 3D culture of cells of defined physical dimensions. Permeation of Matrigel or other hydrogel precursors into chromatography or filter paper is done to yield paper‐supported hydrogels (adapted from Ref. 71)

Whitesides group, after observing requirements of specialized engineering approaches and instrumentation in silica/glass and polymer‐based substrates, came up with a relatively simple and cost effective approach, that is, paper‐based microfluidic systems.^71^ In this technique, chromatographic papers are used to pattern hydrophobic barriers by wax printing. Then, suspensions of cells are impregnated on the papers. Multiple papers are stacked over each other to mimic the 3D architecture. These papers can later be destacked for layer‐by‐layer molecular analysis.^72^ Several reports suggest culture of cancerous and endothelial cells by this fashion to validate their proliferation profiles.^73^ Recently, paper‐based devices have been compared with 3D spheroid culture of MDA‐MB 231 cells. Whatman filter papers were used to pattern 96 multilayer array consisting cells and were further tested against different cell based assays to provide information regarding their migration (Figure 5).^71^ Spheroids and stacked paper‐based 3D culture of cells provided a comparative evaluation of cell density, complex gradients, and proliferation.

## 6 Matrices for microfluidic 3D culture systems

Matrix utilized to support 3D culture, also known as *Scaffold*, plays a pivotal role in assuring better and reproducible growth, differentiation and cell‐to‐cell signaling. Microfluidic technology has utilized gel‐based and gel‐free matrices for various applications in the field of biology.

### 6.1 Gel‐supported culture

Mass transport is the concerning factor in the development of effective 3D culture, which is addressed by perfused microfluidic‐engineered scaffolds.^74^ Of particular interest, hydrogels hold great potential in the development of complex and clinically relevant 3D cellular architecture.^74^ The scaffold must promote healthy development of cells, through the transport of respiratory gases, essential nutrients exchange, as well as be amenable to changes in shear‐stress when being optimized for structural features. Extracellular proteins such as collagen, fibrin, hyaluronic acid, Matrigel, fibronectin, agarose, poly (ethylene glycol) diacrylate, or mixture of the aforementioned have been used to support healthy development.^75^ Such factors, hyaluronic acid and collagen, have been used to promote the growth of endothelial cells to study the influence of VEGF on their proliferation and migration.^75^ The concentration, perfusion, and diffusion rates were comfortably monitored via microfluidic channels, allowing for easy access to manipulate and study the cells.

Hydrogels provide a number of optimization parameters such as pore size, fiber thickness, gradients, and cell seeding which can be manipulated to develop a robust 3D culture system.^76^ Sung et al. demonstrated that collagen fibers of different thickness could easily be obtained by controlling the polymerization of collagen matrix, done by varying pH and preincubation temperatures.^77^ Human mammary fibroblast cells were cultured in collagen matrix of differently thickened fibers and the cells were found to exhibit enhanced viability and more stress fiber formation in thicker collagen fiber systems as compared to the thinner ones.^77^

With microfluidic technology being capable of producing scaffolds of different shapes and dimensions, along with the hydrogel‐based parameters, allow changes that more mimic the microenvironment and structures found in vivo. Hwang et al. developed poly (lactic‐co‐glycolic acid) (PLGA) microfiber shaped scaffolds within a PDMS microfluidic chip for tissue engineering purposes.^78^

The 3D encapsulation of cells within hydrogels has also been investigated for the development of tissue engineering constructs. Burdick et al. developed functionalized polymeric hydrogels such as polyethylene glycol or hyaluronic acid for the encapsulation of stem cells in a 3D fashion. Although the hydrogels were optimized for cytocompatibility and minimum processing steps for hydrogels, some adjustment to analysis protocols are further required to validate the systems.^79^

In addition, multiple hydrogel layers can be created by the use of laminar flow. Kunze et al. demonstrated culture of neurons in a multilayered agarose‐alginate scaffold comprising of four inlet channels in a PDMS microfluidic chip.^80^ The neural layers thus generated were more realistic and close to their native counterparts.

### 6.2 Gel‐free systems

**Figure 6:**
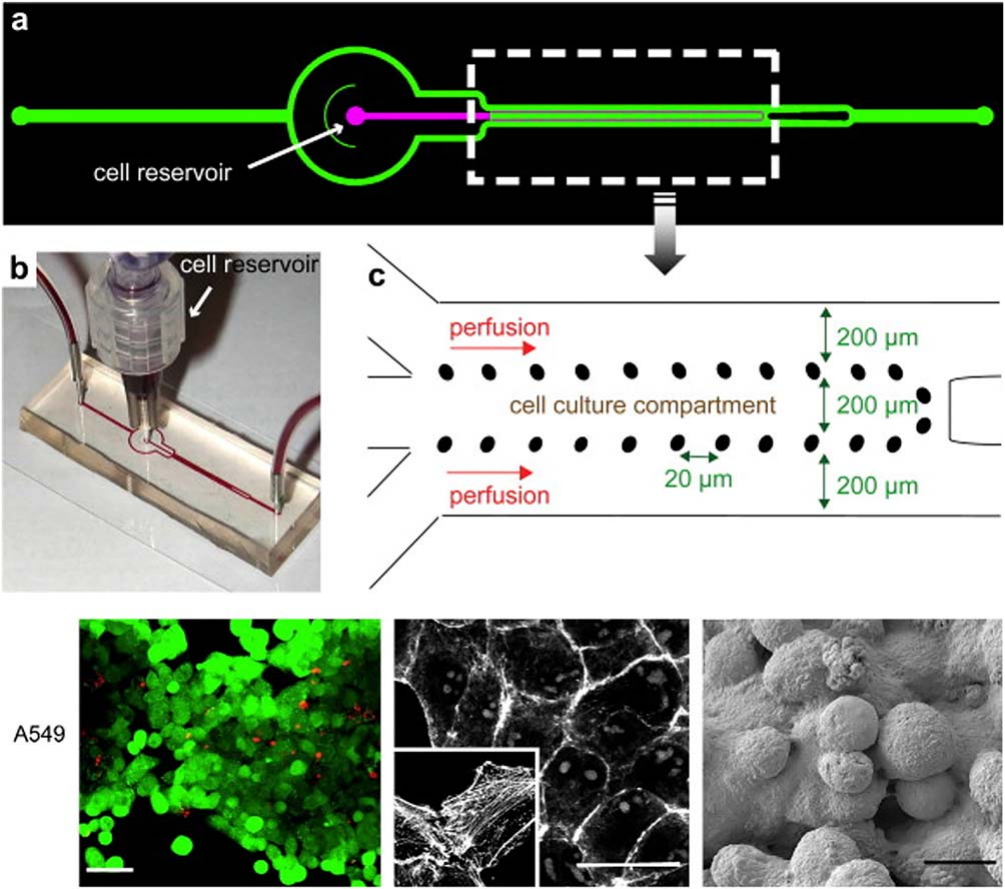
Gel‐free 3D microfluidic cell culture system for A549 cells. (a) The system has two inlets (one for culture medium infusion, one as cell reservoir) and one outlet. (b) Prototype and (c) dimensions of the system (adapted from Ref. 81)

Hydrogels often vary in their composition and properties which limit the transport of nutrients and oxygen through thick and dense hydrogels thus leading to possibilities of reduced viability of cells within 3D culture systems.^11^ Efforts have been made to get rid of gel‐based culture systems to tackle these issues (Figure 6). In one such attempt, polyethyleneimine‐hydrazide, an intercellular polymeric linker, has been used to culture human cancerous cells.^81^ Cells were modified for better interaction with hydrazides which led to aggregation of cells without the use of hydrogel matrices.

Microwells for the 3D culture of cells have been employed to decrease the dependency on gel based matrices.^70^ In microwells, cells are perfused from bottom of a polycarbonate‐based well and medium is supplied upward through the culture wells.

Spheroids can also be cultured in a 3D format using gel‐free systems by employing microfluidic approach.^82^ A 3D metastatic prostate cancer was modeled by coculturing cells in a microfluidic device. For this, hanging drop method was modified and tumor spheroids were cultured inside PDMS microbubbles. Microbubbles were produced by gas expansion molding of PDMS.^83^ Cells were captured inside them and surprisingly viable for more than 5 days. These spheroids were further tested for cytotoxicity against doxorubicin.

Dielectrophoresis has been used to develop a new type of gel‐free culture of cells that utilizes a combinatorial approach of cell sorting and in situ assembling. Schutte et al. cultured hepatocytes and endothelial cells in a sinusoid‐like 3D fashion and found that only viable cells were guided by dielectrophoresis into cell‐assembled gaps preconditioned with collagen.^84^

Cell immobilization without the use of hydrogels can be done by incorporation of polyelectrolyte complex coacervation in the micropillar bound channels. Choi et al. showed culture of human adipose tissue‐derived stem cells by precoating the micropillars with fibronectin and hence growing neurospheres without the aid of hydrogels.^85^

## 7 Applications of 3D cell culture

### 7.1 Tissue engineering: organ‐on‐a‐chip technology

**Figure 7:**
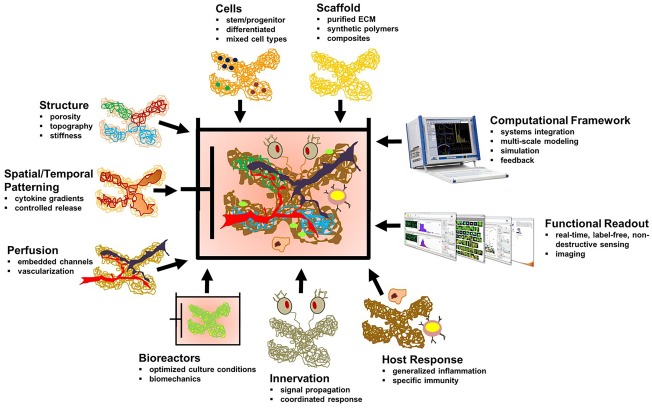
Different components of 3D cell culture for tissue engineering. A perfect combination of cells, scaffold and continuous perfusion with adequate vascular supply and host responses along with functional readout is required to develop tissue/organ substitutes

**Figure 8:**
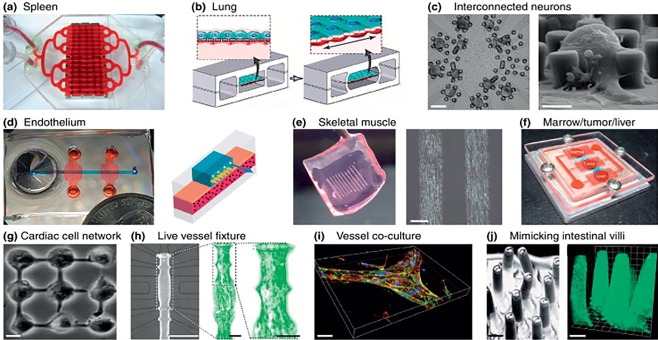
Different human organs microfabricated on chip. (a) Spleen. (b) Lung. (c) Neurons. (d) Endothelium. (e) Skeletal muscle. (f) Marrow/tumor/liver. (g) Cardiac network. (h) Vessel. (i) Vessel co‐culture. (j) Intestinal villi (adapted from Ref. 86)

Tissue engineering focusses on development of tissues/organ substitutes that maintains/restores/improves the functioning of a tissue or whole organ (Figure 7). Microfluidics technology has emerged as a robust platform for tissue engineering.^86^ Vukasinovic et al. developed a microfluidic perfusion device for regenerative medicine that permits growth of tissue equivalents within dynamically controlled environments reproducibly. Gottwald et al. successfully developed chip‐based platform for the culture of hepatocytes, embryonic cells, and stem cells in well‐organized tissue‐like manner. Whitesides et al. integrated microfluidics in tissue engineering and developed 3D tissue constructs artificially. They used PDMS to synthesize modular tissue constructs at high cell density. Stelzle et al. tested liver toxicity by growing real liver‐like tissue comprising of coculture of hepatocytes and endothelial cells in a microfluidic chip. Ethanol toxicity was successfully tested in this pseudo liver. Organ‐on‐a‐chip is a technology that simulates mechanics, functions, and physiological responses of entire organs in a 3D microfluidic cell culture system.^86^ For the first time, it became possible to model human organs in vitro (Figure 8, Table 1).

1. *Lung‐on‐a‐chip*: Wyss institute led by Ingber and coworkers have developed a Lung‐on‐a‐chip system which mimics actual alveolar‐capillary interface on a chip.^87^ Alveolar epithelial cells and endothelial cells were cultured on the opposite sides of a thin, flexible, porous, and ECM‐coated PDMS membrane. This chip was used to study the responses of lung to various bacteria and inflammatory mediators such as cytokines.^88^ It provides a cost‐effective alternative to preclinical models and has been used to screen a number of drugs. Toxicity of silica nanoparticles were evaluated on this biomimetic microsystem. Similarly, liver, kidney and adipose tissues have been also modeled by microfluidic chip technology for the better prediction of drug responsiveness prior to preclinical and human studies.^89^
2. *Intestine‐on‐a‐chip*: Drugs given via oral route are generally absorbed in the small intestine. This process is imperative during the development process of drugs or chemicals, so as to be able to evaluate drugs’ absorption, distribution, metabolism, elimination, and toxicity.^90^ Small intestinal region is lined with enterocytes and goblet cells. Kimura et al. developed an intestinal model with a membrane and vascular flow simulating the epithelial barrier and the epithelial‐endothelial barrier.^91^ Mahler et al. came up with a microscale cell culture analog of gastrointestinal tract with digestion functionality and mucus layer along with realistic cell populations.^92^ These chips were utilized to screen drugs for several GIT diseases and the results obtained were used to correlate preclinical or clinical experiments.
3. *Liver‐on‐a‐chip*: Most of the drugs are withdrawn from research pipeline because of severe dose‐related toxicity, especially liver toxicity, that is, hepatotoxicity. While in vitro models exist for identifying drug‐induced liver toxicity, their utility is drastically limited. Therefore, it is the need of hour to develop an efficient, reliable, accurate, and inexpensive tool for testing liver toxicities.^93^ Microfluidics has shown potential to solve the problem by offering on‐chip liver tissue models which can maintain metabolic activity and phenotype of the poorly viable hepatocytes. Khetani and Bhatia developed a multiwall micropatterned coculture system comprising of hepatocytes along with endothelial cells, stellar cells, Kupffer cells, and fibroblasts.^94^ This chip was able to maintain phenotypic functions for several weeks. It also simulates the morphology of lobules to provide hepatocyte functionality. They performed 9‐days experiment on this chip to test the repeated dose toxicity of troglitazone. Recently, Midwoud et al. integrated intestinal and liver slices into different compartments of a microfluidic device with sequential perfusion between the compartments to study the interorgan interactions.^95^
4. *Tumor‐on‐a‐chip*: Cancer therapeutics require selective killing of cancer cells while leaving the normal ones unaffected. Recently, several 3D tumor tissue models have been developed to mimic cancerous tissues.^96^ Spheroid culture in vitro assists in HTS of single chemotherapeutic agents as well as large combinatorial arrays of drug cocktails. Microfluidic devices enable performing HTS with in the same chip with very minute amount of reagents as compared to typical multiwall plate experiments.^97^ Also, continuous perfusion provided by microfluidic chips resembles the heterogeneous blood supply to tumor tissues.^98^ Jang et al. developed a microfluidic system possessing a capacity to generate an array of drug concentrations (∼100) and cocktails. This helped in fast screening of a number of drugs at various concentrations and IC_50_ at different time points were easily determined which were in agreement with earlier published studies.^99^ Jedrych et al., in a different study, tested different concentrations of a photosensitizer on the viability of lung cancer cells, A549.^100^
5. *Vessels‐on‐a‐chip*: Blood vessels are highways and subways of the body involved in most of the medical conditions. The challenge is to grow vessels and capillaries similar to different microenvironments in vivo.^101^ Dike et al. and Kobayashi et al. demonstrated growth of multiple cells such as endothelial and smooth muscle cells on microfluidic chips which formed capillary tube‐like structures. However, mechanical properties of cultured capillaries still need further optimization.^102^ The other major challenge in microfluidic culture of vascular cells is to mimic the microenvironment surrounding the vessels. Chung et al. showed that cancer cells attract the endothelial cells to form capillaries whereas smooth muscle cells suppressed them to do so.^103^ Song et al. developed a microfluidic device to culture breast cancer cells and evaluate the effects of chemokines.^104^ Gunther et al. demonstrated capability of microfluidic chip to allow long‐term culture of cells with functionality of controlled delivery of drugs to them.^105^
6. *Body‐on‐a‐chip*: Existing microfluidic chips represent single organs and hence are unable to predict the effects of drugs on the whole body. Multiorgan‐on‐a‐chip was designed to address these issues.^106^ Different compartments containing cells from different organs were cultured and connected based on their real sequence to assess the systemic effects of drug action and metabolism in different organs.^107^ This chip was developed based on a mathematical pharmacokinetics‐pharmacodynamics modeling. 5‐Fluorouracil was tested for its toxicity against different organs such as intestine, adipose tissue, liver, and lungs. 108 This mechanism has really shown good potential to replace animal models for preclinical testing of drugs.^89^ Pharmacokinetic profiles of drugs can also be evaluated by coculturing different cells in microfluidic 3D systems. Baker et al. determined drug absorption in an integrated microscale cell culture analog which was based on simplified mathematical representation of the human body.^109^ The system consisted of interconnected compartments with specified flow parameters in which liver, lung, and adipose cells were seeded and cultured in fluidically linked fashion. The medium circulated similarly to how blood does and the systemic effects of drug were evaluated. This microfluidic chip had limited crosstalk between cell culture compartments which reflects the in vivo conditions where different organs have their own particular environments.

**Table 1:** Summary of applications of 3D cell culture with reference to organ‐on‐a‐chip technology, 3D cellular aggregates and tissue models for the development and characterization of nanoparticles

**Tissue engineering: Organ‐on‐a‐chip technology and applications**			
**Organ‐on‐chip**	**Cells used**	**Applications**	**References**
Lung‐on‐Chip	Alveolar epithelial and Endothelial cells	Responses to bacteria and cytokines; toxicity study of silica nanoparticles	87, 88, 89
Intestine‐on‐Chip	Enterocytes, Goblet cells	Absorption, distribution, metabolism, elimination and toxicity studies; microscale analog of the GI tract	90, 91, 92
Liver‐on‐Chip	Hepatocytes, Endothelial cells, Stellar cells, Kupffer cells, Fibroblasts	Maintained phenotypic functions and simulated morphology of lobules; toxicity testing	93, 94, 95
Tumor‐on‐Chip	Tumor Spheroids	HTS screening of single and combinatorial arrays	96, 97, 98, 99, 100
Vessels‐on‐Chip	Endothelial and Smooth Muscle cells	Growth of microvasculature; studying the effects of chemokines	101, 102, 103, 104, 105
Body‐on‐Chip	Slices of whole organs	Studying effects of drugs on multiple organ systems	89, 106, 107, 108, 109, 110
**3D culture of cells and their applications**			
**Cells**	**Features and studies**		**References**
Cardiac	Enhanced cardiomyogenic differentiation; extended study of phenotype; morphology, and cellular viability		111
Liver	Maintained phenotypic quality of liver cells; predictive in‐vivo toxicity		69, 112
Stem	Controlled differentiation due to precise stimuli; migration and morphological change studies		85, 111, 113
Neural	Extended viability with a perfusion of oxygenated media; electrophysiological, viability, and biosensor studies		114, 115
Cancer	Invasion and migration studies; more responsive drug study mimicking 3D microenvironments		46, 82, 116, 117
**3D tissue models for nanoparticles’ development and characterization**			
**3D tissue models**	**Major studies and applications**		**References**
Blood vessels	Interaction of injected nanoparticles in systemic circulation; endocytosis and shear‐responsiveness of particles; targeting efficiency		114, 118, 119, 120, 121, 122, 123, 124, 125, 126, 127
Lungs	Alveolar‐capillary interface model with mechanical breathing motion; translocation and toxicity of silica nanoparticles		89, 128, 129
Liver	Primary hepatocytes‐based 3D spheroidal platform; high throughput clinical screening and metabolic studies of nanoparticles; toxicity studies		69, 94, 130, 131, 132, 133, 134, 135, 136, 137
Tumor	3D tumor structure with dynamic flow conditions; influence of size and surface modification of nanoparticles on transport, penetration, and accumulation		138, 139, 140
Heart	3D cardiomyocytes‐based cell sheets with contractile functionality; real‐time calcium dynamics in hypoxic conditions		141, 142, 143

Hsieh et al. developed a microfluidic cell culture platform with integrated microheaters, temperature sensors, and micropumps for real‐time examination and assessment of cellular functions. There were five reservoirs for medium or drugs in the cell culture module, which were delivered via microchannels to a thin microculture chamber. Mitotic activity and the interactions between oral cancer cells and anticancer drugs were investigated.^110^ However, there is still a long way to go to come up with a perfect body‐on‐a‐chip system.

### 7.2 Different types of cell culture and relevant applications

A 3D culture of cells of different origins has been successfully established and employed for various applications as discussed below:

1. *Cardiac cells*: Diseases related to heart are one of the major causes of deaths worldwide and hence investigating cardiac cells for the development of new treatments is crucial. Wan et al. studied the differentiation of murine embryonic stem cells into cardiac myocytes in a PDMS microfluidic device and found that 3D culture enhanced cardiomyogenic differentiation as compared to conventional well‐plate cultures. Vunjak‐Novakovic developed a coupled system consisting of an array of micro‐bioreactors and microfluidic platform. They cultured rat neonatal cardiomyocytes to form spatially uniform layers and investigated phenotypes, morphology, and cellular viability for an extended period of time.^111^
2. *Liver cells*: Liver is the major organ for the metabolism of drugs and this along with evaluation of hepatotoxicity is important in the development of new therapies. It has remained a challenge to maintain the phenotypic quality of liver cells in vitro. Microfluidic technology with 3D culture changed the whole paradigm of understanding liver diseases and drug metabolism. Yu et al. showed in a microfluidic 3D hepatocyte chip that in vitro hepatotoxicity testing has the potential to accurately predict in vivo toxicity.^69^ Similarly, Leclerc et al. developed a microfluidic biochip‐based toxicogenomic analysis of 3D cultured HepG2/C3A cells.^112^
3. *Stem cells*: Stem cells that can be artificially grown and differentiated into cells specific to certain tissues have a very promising potential in the development of futuristic therapies.^113^ Conventional 2D cultures were unable to control the differentiation patterns of stem cells precisely as a particular set of stimuli guides the whole process. Microfluidic technology has this feature. Kang et al. developed a gel‐free 3D culture system for the culture of human adipose tissue‐derived stem cells. A low oxygen gradient was provided in this culture which activated the Wnt5A/β‐catenin signaling cascade and led to self‐renewal and transformation of stem cells into neurons.^85^ Vunjak‐Novakovic and coworkers utilized 3D culture system to study the cell‐cell interactions human mesenchymal stem cells and HUVEC.^111^ They developed this coculture in a spatially controlled 3D fibrin hydrogel system. They found that stem cells show strong distance dependent migration toward endothelial cells and formed a stable vascular network eventually.
4. *Neural cells*: Neurons play an important role in the signal transduction throughout the brain system and this property has been harnessed to study various neurological disorders such as Alzheimer and Parkinson disease. Neural cells can act as drug testing biosensors because of their specific binding affinities with drugs and toxins. Wheeler et al. studied electrophysiological properties of neural cells in a 3D microelectrode array. This system comprised of individually patterned thin films that formed a cell chamber conducive to maintaining and recording the electrical activity of a 3D mesh of neural cells. They found that cells were more viable in this system and further tested for responses against tetrodotoxin.^114^ Culture of brain slices is plagued with necrotic problems. Potter and coworkers developed an interstitial microfluidic perfusion system for supplying oxygenated nutrient medium to brain slices and found that they were viable and functional even after 5 days in vitro while maintaining the in vivo architecture.^115^
5. *Cancer cells*: Cancer is a fatal disease where the cells invade local tissues and metastasize to other vital organs via circulation. Microfluidics along with 3D culture of cancer cells unveil the complexities of cells, their interactions with drugs and hence provide a promising platform to develop novel cancer therapies.^46^ Sung et al. developed a 3D microfluidic system to study the invasiveness of ductal carcinoma cells.^116^ Liu et al. investigated the role of carcinoma‐associated fibroblasts in cancer invasion using a microfluidic 3D cell coculture.^144^ Additionally, microfabricated platforms have been used significantly for anticancer drug screening. Agastin et al. developed microfluidic array systems to culture Colo 205 cells for the purpose of drug screening and toxicity testing.^82^ They used PDMS microbubble system to develop tumor spheroids and tested doxorubicin, and found that cancer cells showed a threefold increase in resistance to drug as compared to when cultured as 2D monolayer cells. Buchanan and Rylander critically reviewed various developments and future applications of microfluidic culture models to study tumor progression and therapeutic targeting.^117^ They found that integration of 3D culture and microfluidic technology has enabled the researchers to develop cancer tissue models mimicking native 3D microenvironments. Significant progress has been made in the high throughput drug screening by using tumor‐on‐chip microdevices.

### 7.3 3D tissue models for nanoparticles’ development and characterization

Field of nanomedicine or nanoparticles based therapeutics has seen significant spur of advancements mainly focused on development and characterization of customized carrier systems specifically designed to deliver payload of active molecules and diagnostic agents for sustained, pre‐programmed and/or targeted applications.^145^, ^146^, ^147^ An ideally designed nanoparticulate system, with size range of 50–200 nm, can provided prolonged circulation time, efficiently translocate across cellular membranes and unload encapsulated actives at desired site in a programmed manner to minimize off‐target adverse effects. Further, the innovative materials with varied chemical make‐up employed in fabrication of nanoparticles allow encapsulation of drugs and diagnostic agents with diverse physicochemical properties.^148^, ^149^

Despite such substantial and alluring advantages that nanoparticulate carrier systems have to offer, the “bench‐to‐bedside” transition of these nanotechnology based formulations has remained very limited so far.^150^ This less than impressive commercial success for such advanced nanocarriers could potentially be attributed to the challenges faced during their characterization and subsequently in their bulk scale consistent production. Characterizing the performance of nanoparticles at initial stages of development involves use of conventional in vitro models, mainly 2D cell culture models. Attempts of transition from such over‐simplified models which generally provide over‐promising outcomes face significant difficulty in verifying results of efficiency and performance of nanoparticles in more complicated in vivo settings. Information regarding cellular interaction of nanoparticles can be collected using in vitro cell culture models, whereas data related to efficacy and toxicity of nanoparticles can be obtained using animal models. However, there exists a gap in information, pertaining to interactions of nanoparticles with tissue structures and components such as cells, ECM and other physiological factors, which could be explored using 3D tissue models. Further customization of such intermediate models to account for variations in phenotypic expressions and concentration gradients in both healthy and pathological environments, can provide more appropriate settings for assessing toxicity, efficacy, and targeting efficiency of nano drug carrier systems.

The scope of microfluidic technology for consistent bulk manufacturing of nanoparticles has been well recognized.^151^, ^152^ Combining the technologies of tissue engineering and microfluidics has potential to create physiologically relevant 3D models for efficient development and characterization of nanoparticles and has ability to fill in the gap that exists between the outcomes obtained from conventional in vitro models and that from in vivo models. Section below describes the key advancements made in this direction using microfluidics.

1. *Blood vessels*: To investigate the interaction of intravenously injected nanoparticles in the systemic circulation, most importantly their transport, accumulation pattern, and toxicity, researchers have developed specific 3D models mimicking various vascular features such as geometry (straight channels^114^, ^118^, ^119^ and bifurcations^120^, ^121^, tortuosity, and shear stress^122^ using microfluidic technology. Particle accumulation within the vasculature was observed to be size dependent, based on another model,^123^ where microparticles preferred to localize on the margins in comparison to their nanosized counterparts. Particle interactions with blood component and their influence on activation/aggregation of platelets have also been explored using a microfluidic channel‐based 3D vascular model.^119^ Further, the endocytosis of particles and shear‐responsiveness of programmed particles for targeted delivery have been investigated in microfluidic chips designed to mimic variation in shear stress in different vascular regions.^121^, ^124^, ^125^ Recent advancements show promise to make this technology available for both rapid and economical^126^ high‐throughput screening and characterization of injectable nanoparticles, and to investigate their permeability behavior^114^ and targeting efficiency.^127^
2. *Lungs*: The pulmonary route is one of the most investigated routes for drug delivery using nanoparticles for both local and systemic ailments.^128^ The inability of 2D cell culture models to generate the complexity of the human lungs has forced researchers to look toward microfluidic technology that can incorporate biological, structural, and mechanical intricacies of the lungs into a 3D model. A biomimetic alveolar‐capillary interface model with mechanical breathing motion, when used to study translocation and toxicity of silica nanoparticles, showed enhanced free radical production and increased adhesion molecule expression.^129^ Besides such ventilation‐perfusion based lung‐on‐a‐chip model, another study evaluated gelatin microparticles containing TGF‐β1 in a multicompartment 3D microfluidic model, representing key organs including the lungs, and demonstrated possible cross‐talk which takes place between organs in the body.^89^
3. *Liver*: Due to limitations of animal models in predicting hepatotoxicity of new drugs or nanoparticles owing to their differences with human physiology, extensive attempts have been focused on developing in vitro 3D tissue engineered and microfluidic models capable of mimicking functionality as well as both healthy and disease microenvironments of liver.^130^, ^131^ Studies reported that use of primary hepatocytes‐based models, designed to maintain cell activities for longer period of time, in assessing nanoparticles produce results that better correlate with in vivo settings.^94^, ^132^ For high‐throughput clinical screening and metabolic studies of nanoparticles, 3D hepatic spheroidal platforms have been extensively explored.^133^, ^134^ To study toxicity of nanoparticles in dynamic flow conditions, microfluidic models such as liver‐on‐a‐chip have also been developed.^69^, ^135^ A step further, several studies have also reported multiorgan microfluidic models to investigate prodrugs that first metabolized in the liver‐mimicking section before reaching to the other organs on a chip.^136^, ^137^
4. *Tumor*: The multifaceted variations, specifically the vascularization and lymphatic access, of tumor physiology^138^ in comparison to a normal tissue pose an array of additional challenges in development of efficient and targeted nanoparticulate drug delivery systems which could overcome perilous side effects which are associated with most anticancer drugs to healthy tissues. For improved characterization and optimization of nanoparticles’ transport behavior in intricate tumor structure and dynamic flow conditions, a microfluidic model incorporating 3D tissue engineering can be utilized.^139^ A tumor‐on‐a‐chip model was designed to mimic physiological conditions in a study where influence of size and surface functionalization of gold nanoparticles on their transport, penetration, and accumulation in tumor tissue was investigated in real‐time.^140^
5. *Heart*: Attempts have been made to mimic heart physiology for use in drug discovery and development process using advancements in tissue engineering and microfluidics.^141^, ^142^ Incorporating such technologies with employment of cardiomyocytes cell sheets have resulted in more physiological relevant cardiac model with contractile functionality.^143^ Another study, with use of two primary rat myocyte cell sheets draped together, demonstrated concurrent impulsive beating after 7 days. A microfluidic model was also reported with capability of generating hypoxic conditions in the cardiomyocytes and observing real‐time calcium dynamics in these cells. Still, much research and efforts are required to create 3D microfluidic models with improved representation of cardiac functionality and physiological microenvironment for characterization of nanodrug carriers.

Overall, the use of 3D tissue engineered microfluidic platforms represent an innovative step forward to make high‐throughput drug screening and characterization of nanocarriers both faster and inexpensive, while generating information that better relates to human physiology in comparison to conventional in vitro or preclinical animal models. Nevertheless, several key challenges still need to be overcome to fully incorporate different biological, structural and mechanical features and complexities of an organ in such models. Collective efforts are required to focus on developing a whole body‐on‐a‐chip model capable of reproducing both normal and pathophysiological variations. Although some progress has been made in this direction so far, a lot of work is still needed to fully explore and realize scope and applicability of such technology in studying drugs and nanoparticle for better clinical translation.

## 8 Future Perspectives

Combination of microfluidics and 3D cell culture has great potential to provide efficient methods for biomedical applications, tissue engineering, and drug screening in physiologically relevant micromilieu. However, there are many challenges which need immediate attention. For instance, there is limited access to the cultured cells in microsystems which further becomes tough and complicated while sampling. This requires development of dedicated methods and devices for functional studies and screening. Even after this, commercialization of mature and ready‐to‐use devices, and making them available to scientists is challenging because of the technical hurdles. In this review, we have described different approaches and techniques for microfluidic 3D cell culture, each of them has its own strengths and weaknesses with respect to mimicking the various aspects of 3D culture. For example, there are a number of methods for 3D spheroid generation but discrepancies occur as different cell lines behave differently when cultured using the same method. Transition from 2D to 3D not only adds one more dimension in terms of shape and structure but also in terms of acquired data, that is, high content imaging of a 3D model acquire stacks of images at high resolution at higher speeds, and hence increasing by a thousand to a hundred thousand the data acquired during one single experiment. So, new ideas and methods must be considered to improve and build on the current drug development process, and achieve success. In the near future, we expect new research in microfluidic 3D cell culture to extend in two directions mainly to improve its robustness and parallelism and to facilitate readout. The first one should be the development of automated, high throughput, reproducible, reliable, cost‐effective, and easy‐to‐use microfluidic 3D cell culture systems. The second direction should be the smooth and hassle‐free integration of complicated microfluidic systems holding great in vivo relevance. Furthermore, it is highly expected coming era will see new developments and discoveries in the field of tissue engineering with the advances in microfluidics cell culturing techniques.

## 9 Conclusions

Undoubtedly, 3D culture is a blessing for scientists but there are few issues that if addressed, could change the whole perspective of scientific community. Complexity associated with access to cultivated cells and further sampling for assays is a big problem with microfluidic based 3D culture systems. The current systems lack control of dynamics and spatial presentation of various signals, which requires meticulous attention. There is also a strong need of cost effective and easy‐to‐use systems as technical issues cast a dark shadow over this novel and fruitful technology. Although, organ‐on‐a‐chip and human‐on‐a‐chip have drawn attention, integration of complicated microsystems that can closely mimic the in vivo environments still need further optimization. A perfect combination of bioinformatics, systems biology, and engineering may help in overcoming these challenges.
